# Effectiveness of Scenario-Driven Virtual Reality for Improving Nursing Students’ Behavioral Responses Toward Older Adults With Dementia: Protocol for a 4-Arm Postintervention Study

**DOI:** 10.2196/95684

**Published:** 2026-09-22

**Authors:** Dianis Wulan Sari, Hadziq Fabroyir, Neisya Pratiwindya Sudarsiwi, Manami Takaoka, Nurul Hidayati, Etty Rekawati, Muhammad Rafi Insan Fillah, Widian Sasi Disertasiani, Nauvila Fitrotul 'Aini, Nur Arifah Astri, Gerry Sihaj, Harits Ar Rosyid, Andika Bagus Nur Rahma Putra, Ayumi Igarashi

**Affiliations:** 1 Faculty of Nursing, Universitas Airlangga, Campus C MERR, Surabaya, East Java, 60115, Indonesia, 62 81259497373; 2 Dementia and Aging Care Research Center, Universitas Airlangga, Surabaya, East Java, Indonesia; 3 Department of Gerontological Home Care and Long-term Care Nursing, The University of Tokyo, Tokyo, Japan; 4 Department of Informatics, Institut Teknologi Sepuluh Nopember, Surabaya, East Java, Indonesia; 5 Department of Advanced Gerontological Nursing, Graduate School of Nursing, School of Nursing, Chiba University, Chiba, Japan; 6 Associate Degree Nursing Program, STIKes Satria Bhakti Nganjuk, Nganjuk, East Java, Indonesia; 7 Faculty of Nursing, Universitas Indonesia, Depok, West Java, Indonesia; 8 Faculty of Engineering, Universitas Negeri Malang, Malang, East Java, Indonesia

**Keywords:** aging, dementia, education, health professional, virtual reality, long-term care

## Abstract

**Background:**

Dementia training requires innovative strategies to improve nursing students’ attitudes, knowledge, and behavioral responses regarding ageism. Virtual reality (VR) offers immersive learning, but existing interventions often rely on passive 360° viewing, which provides limited opportunities for practice communication.

**Objective:**

This study aims to evaluate the effectiveness of a scenario-driven, AI-based VR awareness simulation adventure (ASA) in improving nursing students’ attitudes toward people living with dementia, knowledge of dementia, ageism, and intention to help.

**Methods:**

This protocol outlines a 4-arm quasi-experimental 2×2 factorial design involving ASA VR and conventional lecture-based dementia training. Nursing students will complete preintervention and postintervention assessments measuring attitudes toward people living with dementia, dementia knowledge, ageism, and intention to help. The sample size justification is based on previous VR-based dementia education studies that reported large effects (Cohen *d*=0.9). Data will be analyzed using a 2-tailed independent *t* test, paired tests, analysis of covariance, and effect sizes. The intervention will be described in accordance with the TIDieR (Template for Intervention Description and Replication) guidelines. Data management, harm monitoring, and ethical approval procedures are specified.

**Results:**

Data collection began in December 2025 and was completed in June 2026, with 182 participants recruited. Data analysis is currently ongoing. Findings will report on the effectiveness of ASA VR in enhancing attitudes toward people living with dementia, dementia knowledge, ageism, and intention-to-help outcomes.

**Conclusions:**

This protocol introduces Indonesia’s first scenario-driven VR dementia simulation training. Findings may inform the development of culturally adapted VR-based dementia training.

## Introduction

Dementia has become one of the most pressing global public health challenges, with its prevalence increasing rapidly in both high-income and low- and middle-income countries [1,2]. As the number of people living with dementia continues to rise, the importance of dementia care education is growing [3]. There is an urgent need for educational programs that not only provide knowledge about dementia but also foster empathy and promote appropriate support behaviors toward people living with dementia [4].

Our previous program aimed to enhance understanding of dementia through a combination of learning and an immersive experience [5-7]. Participants first studied the basics of dementia, then engaged with the story of a fictional woman named Shizue, who lives with dementia [8]. Her story was presented through a drama and a 360° video, allowing participants to experience it from both a third-person perspective and Shizue’s own perspective. This approach was designed to improve attitudes toward people living with dementia, reduce ageism, and increase intention to help, compared to standard dementia classes [8]. However, a key limitation was the lack of opportunity to simulate actual helping behaviors.

To address this gap, we developed a new interactive virtual reality (VR) component. This program, called the Awareness Simulation Adventure (ASA) VR program, introduces an innovative, scenario-driven, and AI-based learning environment. In this updated program, participants enter a virtual environment based on Shizue’s story and engage in helping actions, such as initiating conversations with her. This allows learners to practice helping behaviors in a realistic and emotionally engaging context. A key strength of our approach is the consistent use of the fictional character Shizue throughout the program. Experiencing dramatized scenes from her life across multiple formats contributes to a coherent and immersive learning experience.

Simulation-based learning theory suggests that effective learning occurs when knowledge acquisition is followed by opportunities to practice behavioral responses in realistic situations [9-11]. While immersive experiences, such as VR, narratives can enhance empathy and perspective-taking, they may not necessarily translate into behavioral competence without opportunities to actively practice helping responses. Interactive simulation environments allow participants to apply knowledge, test decision-making, and reflect on their actions in a safe setting [9,12]. Therefore, integrating immersive perspective-taking with interactive behavioral simulation may provide a more comprehensive educational approach. Building on our previous program [8], the ASA VR program was developed to introduce an interactive simulation component that enables participants to actively practice helping behaviors toward people living with dementia.

This study addresses the following research question: does participation in the ASA VR program improve nursing students’ attitudes, knowledge, and intention to help people living with dementia and reduce ageism?

## Methods

### Study Design

The research will use a 4-arm quasi-experimental 2×2 factorial design based on two factors: (1) previous exposure to VR-based dementia education and (2) current intervention allocation (ASA VR vs standard lecture-based materials) to assess the effectiveness of a scenario-driven VR simulation for dementia training among nursing students. This study is a continuation of our previous research, as described in the referenced papers [6,7]. The study is specifically designed to examine the impact of adding an interactive behavioral training component on learning outcomes, with a focus on fostering empathy and attitudes. Briefly, the previous study used a pre-post design with 2 arms: an intervention group that received a VR dementia education program and a control group that received conventional learning methods. The VR-based dementia education program integrated multiple learning modalities, including 2D videos depicting desirable and undesirable care practices, a lecture-based component, VR-based learning using 360° videos (desirable and undesirable), a card game, and facilitated group discussions. Based on these findings, the present study was subsequently developed. As the newly developed ASA VR is an extension of the previously implemented VR-based program, it is important to examine whether its effectiveness varies with students’ prior exposure to VR interventions. In contrast to the earlier program, which primarily focused on understanding the lived experiences of people living with dementia and fostering empathy, the ASA VR intervention in the present study is specifically designed to train supportive behaviors through direct practice of communication and decision-making. Therefore, participants will be categorized into 4 groups according to two criteria: (1) prior participation in a VR-based dementia education program and (2) receipt of either the newly developed ASA VR intervention or standard lecture-based materials in the current study.

This 4-group allocation allows us to test two primary hypotheses: (1) the scenario-driven ASA VR intervention, which emphasizes active behavioral training, will result in greater improvements in attitudes toward people living with dementia, dementia-related knowledge, ageism, and intention to help compared with standard lecture-based materials; and (2) students who have previously participated in a VR-based dementia education program designed to foster empathy and positive attitudes will demonstrate additional improvements after receiving behavioral training through ASA VR, reflecting a cumulative learning effect. Accordingly, this study design enables examination of whether empathy cultivated through prior interventions, followed by subsequent hands-on behavioral training, provides added benefits compared with each approach implemented in isolation. This factorial design allows for the analysis of both main effects and potential interaction effects.

In the current study, participants will be divided into four groups (Table 1): (1) a previously VR-exposed group receiving VR+ adaptive scenario approach (ASA VR); (2) a previously VR-exposed group receiving standard course material; (3) a previously non-VR-exposed group receiving VR+ adaptive scenario approach (ASA VR); and (4) a previously non-VR-exposed group receiving standard course material.

**Table 1.** Allocation of participants into the 4 study arms.

Study arm	Previous VR^a^ exposure	Current intervention	Intervention component
Group 1	Previously VR-exposed	ASA^b^ VR	AI-driven interactive VR simulation with adaptive dementia-care scenarios (30 min) Postquestionnaire
Group 2	Previously VR-exposed	Standard material	Online class discussion related to dementia (20 min) Postquestionnaire
Group 3	Previously not VR-exposed	ASA VR	AI-driven interactive VR simulation with adaptive dementia-care scenarios (30 min) Postquestionnaire
Group 4	Previously not VR-exposed	Standard material	Online class discussion related to dementia (20 min) Postquestionnaire

a VR: virtual reality.

b ASA: Awareness Simulation Adventure.

ASA VR operationalizes evidence-informed instructional principles: (1) problem-centered, authentic tasks with demonstration and guided practice (Merrill’s First Principles of Instruction); (2) anchored, scenario-based learning to encourage transfer (eg, Jasper-style complex problems); and (3) first-person, immersive perspective-taking that has been shown to strengthen empathy in health care education using VR. ASA VR is organized as a dialog-driven, decision-making-based learning system in which learners engage directly with an AI character representing an individual living with dementia. Each scenario depicts a genuine communication challenge frequently encountered in dementia care, requiring students to identify the situation, select suitable communication techniques, and respond verbally in real time. By positioning students as active agents within these interactions, ASA VR is intended to facilitate the transition from empathy and conceptual understanding to the practice of supportive behaviors. The system evaluates users’ responses, provides immediate feedback, and adjusts subsequent prompts based on learners’ choices, thereby creating an adaptive learning pathway. Together, these elements aim to move learners beyond recognition-level knowledge toward situated judgment and prosocial intention.

### Participants and Setting

The study will be conducted among nursing students at an Indonesian university. This simulation is a scenario-driven, interactive VR learning experience designed to cultivate dementia-competent attitudes and behaviors among health care students. Unlike the passive 360° video used in the previous study, ASA VR requires learners to make decisions, engage in role-play with an AI character portraying people living with dementia, and receive immediate, context-specific feedback. Each session will last approximately 40 minutes.

Participants in this study were nursing students at a Faculty of Nursing in Indonesia, who previously participated in our VR-based dementia education study [8]. This follow-up study uses the same cohort to evaluate the additional effect of the scenario-driven VR (ASA VR) intervention. In the previous study, the total sample comprised 203 nursing students: 110 in the control group (who received conventional lecture-based education) and 93 in the intervention group (who received VR-based education using 360° videos). The previous study demonstrated a large effect in improving attitudes toward people living with dementia [8]. In the current study, these participants will be reassigned to 4 groups based on their prior exposure.

This study will be conducted in a computer laboratory equipped with 20 VR headsets (PICO, Immersive Pte Ltd). Due to the limited number of VR devices, the intervention will be delivered across multiple sessions. For the groups receiving the ASA VR intervention, data collection will be divided into 6 sessions, with each accommodating up to 20 nursing students. Each complete session, including an onboarding tutorial, VR experience play, and a posttest assessment, will last approximately 40 minutes.

### Recruitment

Participants in this study were drawn from individuals who had previously taken part in a VR dementia education program, with baseline data collected as part of that study [8]. A general briefing on the research session will be delivered, and detailed procedural information will be provided shortly before the study begins. A notification regarding the study schedule will be distributed approximately 1 week in advance. After a thorough explanation of the research procedures, prospective participants will be invited to provide written informed consent voluntarily. Those who agree to participate will retain the same identification number used in the previous study and will be notified of the specific time and location of their scenario-driven VR session through a class announcement distributed 3 days before the session. On the day of data collection, participants will gather at the designated location and formally register their attendance by signing an attendance sheet. The principal investigator will provide a comprehensive explanation of all study stages, emphasizing participants’ right to withdraw at any time without consequences. Participation is entirely voluntary. Informed consent will also be reaffirmed electronically at the beginning of the Google Form, and checking the consent box and providing “I agree to participate” will indicate that the participant has read the study information and agrees to participate voluntarily.

### Intervention

The ASA VR program is a scenario-driven, interactive VR learning experience designed to cultivate dementia-competent attitudes and behaviors. Unlike passive 360° video approaches used in previous studies, ASA VR requires active decision-making, real-time communication with AI characters portraying people living with dementia, and immediate, context-specific feedback. This interactive approach operationalizes evidence-based instructional principles, as detailed in the “Study Design” section, to foster experiential learning and strengthen empathetic responses in dementia care situations. The complete intervention session lasts 40 minutes.

The intervention in this study consists of an ASA VR program (Table 2), which will be administered to each participant once. The program will be conducted in 6 separate sessions, each lasting 40 minutes and with 20 nursing students per session. A facilitator will supervise each participant. Facilitators, selected from research team members or lecturers from the health and engineering faculties, will undergo joint training conducted by the Japanese and Indonesian research teams. Intervention fidelity will be maintained through facilitator training, standardized VR procedures, and supervised implementation during all intervention sessions.

**Table 2.** Overview of virtual reality (VR) simulation for dementia training.

Session Component	Duration (min)
Previous study: VR-based dementia education program	
Introduction The principal investigator explained the overall study and the content of the informed consent.	3
Pretest Participants will receive a QR code that provides access to information on informed consent and the online questionnaire.	10
Lecture introduction The introduction outlined who was involved in the research and how the research was conducted.	2
Undesirable drama As they observe a narrative featuring a woman living with dementia, participants reflect on her background and the reactions of those around her.	10
Undesirable VR Participants experienced firsthand how people with dementia were mistreated using VR.	10
Discussion Participants shared their impressions of the experience after witnessing an adverse VR scenario.	5
Lecture 1: what is dementia? The lecture consists of dementia and how people with dementia appear.	10
Desirable VR Participants experienced firsthand how effective treatment and emotional changes can be for people with dementia through VR.	10
Desirable drama Participants identify differences in the emotional experiences of people living with dementia and the reactions of individuals in their immediate surroundings between the 2 given scenarios.	10
Lecture 2: what can we do to support people living with dementia? The lecture consists of the treatment of people living with dementia, person-centered care, and social support.	5
N-impro Students will participate as a group alongside a facilitator. The game is structured with components such as “situation cards,” “answer cards,” and “point cards.”	10
Posttest Participants will be issued a QR code to access the online posttest. In addition, there will be a designated space for participants to provide free-form comments expressing their opinions and feedback on the gaming education program.	10
Conclusion Summary about dementia, treatment of people living with dementia, and social support.	5
Current study: VR simulation for dementia education program	
Introduction of the study The principal investigator provided a comprehensive overview of the study, including the content of the informed consent.	5
VR simulation Role-playing simulation where players interact with an AI that takes on the role of a person living with dementia in a virtual world.	30
Posttest Participants will be given a QR code to access the online posttest. Furthermore, there will be a designated area where participants can offer open-ended comments, enabling them to share their thoughts and feedback on the VR-based dementia education.	5

### Previous Dementia Education Program

#### Overview

The dementia education program developed in the previous study was designed as a multimodal intervention that combined multiple learning approaches to foster empathy and positive attitudes toward people living with dementia [3]. The program did not rely solely on didactic lectures but also incorporated scenario-based film, passive VR experiences using 360° videos, group-based games, lectures, and facilitated discussions (Table 2). This approach was designed to enable participants to compare nonsupportive and supportive care behaviors and reflect on their potential impact on the emotional experiences of people living with dementia [8].

#### Program Components

##### Scenario-Based Film

A short film will illustrate contrasting examples of effective and ineffective communication with people living with dementia, allowing participants to compare approaches and reflect on their implications. The learning objectives will focus on understanding how communication practices influence the emotional well-being of people living with dementia, recognizing the importance of social support, and appreciating the role of a dementia-friendly environment. The film will be projected on a large screen to enhance visibility and increase participants’ engagement during the session.

##### Passive VR Experiences Using 360° Videos

A first-person VR film, translated into Indonesian, will present several scenarios related to the lived experiences of individuals with dementia [6,7]. The VR experience will be delivered through head-mounted displays, specifically Pico G2 4K and Pico 4 headsets. Members of the research team will assist participants in operating the devices and ensure that the VR sessions can be conducted simultaneously. Before the activity begins, participants will receive instructions regarding the potential risks of VR sickness and will be advised to discontinue the experience if any discomfort occurs. The research team will monitor participants throughout the session and will confirm that no adverse events are reported [13].

##### Group-Based Games

For this study, we will use a card game called N-impro. N-impro will be an interactive, card-based learning game originally developed in Japan [14] and later adapted for nursing students in Indonesia [15,16]. In this study, an Indonesian-adapted version of N-impro will be used to enhance students’ understanding of communication strategies and caregiving approaches for persons living with dementia. The game will consist of situation, answer, and point cards, with situation cards assigning participants a role, presenting a dementia-related dilemma, and requiring them to make and justify a decision through discussion. These cards were translated into Indonesian and chosen because they reflect culturally appropriate scenarios and common challenges in dementia care, with each card based on real-life experiences collected through interviews. The activity will be conducted in facilitator-led groups of 5-7 students, with each group discussing 2 to 3 cards for approximately 10 minutes to encourage collective reflection and decision-making.

##### A Lecture and Discussion about Dementia

The instructional content will be presented as a video-based lecture displayed on a monitor. To promote attention and understanding, the material will employ animated cartoons. It will be structured into 2 core sections: “What is Dementia?” and “How Can We Support People Living with Dementia?” Originally developed in Japanese, the lecture will explain dementia by covering its characteristics, symptoms, and progression [3]. The research team translated the video and incorporated Indonesian-language lyrics to improve accessibility for the intended audience. Although the video was originally designed as an educational tool for primary school children in Japan, it will be adapted for nursing students to ensure that the content remains clear, engaging, and easy to retain. Subsequently, a discussion session will be conducted to deepen participants’ understanding and encourage critical reflection on the lecture content.

### Scenario-Driven VR for Dementia Education

The ASA VR was developed using insights from previous studies [6,7,17,18]. The authors refined the study design to align with the participants and the study’s objectives. As shown in Table 1, the simulation training program in this research comprises 4 main components. Based on insights from previous studies, the sequence includes a short film portraying inappropriate communication with people living with dementia, a lecture, N-impro gaming, and scenarios demonstrating appropriate communication practices. Building on this foundation, the current study extends the approach to a VR-based simulation to further enhance dementia education. The current study’s intervention session is designed to be completed within 40 minutes. The simulation training program consists of 4 main elements.

### VR for Dementia Education Program

ASA VR is a research platform developed by the authors of this paper, who are affiliated with Institut Teknologi Sepuluh Nopember. ASA VR is a role-playing simulation in which a player interacts with a nonplayable character (NPC) portraying a person living with dementia in a virtual environment. A rule-based scenario and a large language model (LLM) drive the NPC’s behavior. The players are guided based on the topic of conversation and are expected to communicate with the NPC in accordance with dementia care principles. Any user input that violates these principles will result in a penalty, whereas adherence to them will earn points. The scenarios available in ASA VR include:

1. Forgetting to turn off the water tap: the NPC forgets to turn off a faucet.
2. Wearing mismatched clothing: the NPC loses its sense of fashion coordination.
3. Getting lost on the street: the NPC forgets directions and loses awareness of its surroundings.
4. On the way home after buying bananas, the NPC forgets that they already have an excess supply of bananas.
5. Forgetting a recent karaoke outing: the NPC forgets a recent activity in which they participated.
6. Forgot that they had breakfast: the NPC is confused about why breakfast has not been served yet.

Consider the following example scenario: “An older adult appears confused and believes she has not eaten breakfast, despite evidence of a used plate in the kitchen. Responses that acknowledge her feelings, use respectful language, and gently support recall (eg, calmly asking whether she may have eaten earlier or offering to check the kitchen together) are awarded additional points, as they reflect empathy and person-centered communication. Neutral responses that provide information without emotional engagement result in no change in score. In contrast, responses that express irritation, blame, or ridicule the older adult for forgetting (eg, emphasizing her age or accusing her of pretending) lead to point deductions, as they demonstrate ageist and non-supportive behavior*.*”

The ASA VR program is played on the PICO device, which is equipped with the default PICO controller. It is a simple conversational simulation in which players can walk and turn in the virtual environment using the joystick and speak by pressing the “A” button. Players take turns speaking after a robotic assistant signals the start of a conversation. Verbal interactions between a player and an NPC are evaluated based on both the textual content and the amplitude of the spoken voice.

The VR program itself constitutes an active component of the intervention. Participants engage in scenario-driven simulations in which they interact with virtual older adults exhibiting various dementia-related behaviors. In addition to the VR equipment and software described above, administrative materials include electronic and paper-based informed consent forms, participant identification codes to maintain confidentiality, and attendance sheets for session documentation. The ASA VR software and detailed protocols are available upon request from the corresponding author.

### AI-Based Conversational Component

The ASA VR application is built in Unity and uses the OpenAI API through an OpenAI–Unity plugin. Three main functions work together: whisper-based speech-to-text converts what the participant says into text; a GPT-4o chat completion model generates context-aware replies for the NPC based on the dialogue history; and text-to-speech converts those replies back into spoken dialogue. The design is hybrid: the exact wording of each NPC response is generated in real time by the LLM, but conversations are still guided by 6 predefined dementia care scenarios and structured conversational stages. A rule-based conversation manager in Unity controls scene transitions, scoring, and conversation limits. If the LLM detects that a user’s input is irrelevant or off-topic, an in-VR assistant redirects the participant back to the scenario, keeping interactions focused on the communication goals for dementia care. The LLM also labels each user response as “good,” “bad,” or “out-of-context,” and these labels feed a rule-based scoring system that tracks a heart-based score and applies penalties for inappropriate tone. To test the reliability of this automated scoring, the LLM’s classifications were compared with independent ratings from expert dementia care educators for a sample of recorded interactions; agreement measures (Cohen κ and percentage agreement) showed substantial alignment between the system and expert raters when distinguishing “good” from “bad” responses.

Strong emphasis is placed on privacy and data protection. User speech is processed in memory only during the interaction, and no raw audio is stored. After each session, only anonymized text transcripts without direct identifiers are saved, and participants are represented in the system by pseudonymous study IDs. The OpenAI configuration is set so that customer data is not used to train OpenAI models, and Cognitive3D (Cognitive3D, Inc) handles exported analytics via encrypted communication and access controls. All participants provided written informed consent that clearly explained the use of an AI-based conversational agent and the possibility that anonymized interaction data may be reused for research and educational evaluation.

### Control Group

In the previous study, the control group received dementia education through the standard campus curriculum covering dementia knowledge, symptoms, and care. Although the content was similar to that of the intervention group, it was delivered via prerecorded videos on the university’s learning management system [8]. In this study, the control group (Table 1, arms 2 and 4) will receive approximately 20 minutes of online dementia-related discussion and will not participate in the ASA VR intervention. All participants will complete postintervention questionnaires to assess the study’s primary and secondary outcomes. The control sessions will be led by the PI and coauthors from various health care disciplines.

### Measurement

#### Overview

Previous research has evaluated the effectiveness of educational programs in improving attitudes, knowledge about dementia, and intentions to help people with dementia. In line with previous studies, the current VR program aims to deepen experiential learning while enabling the measurement of changes in attitudes and helping behaviors in nursing students’ responses to older adults with dementia [6,7,15,19].

This study evaluated the preintervention and postintervention outcomes of an ASA VR simulation designed for dementia education. The objective is to determine the simulation’s effectiveness by assessing participants’ attitudes toward people living with dementia, their intentions to provide assistance, their dementia-related knowledge, and the stigma associated with dementia. It is hypothesized that engagement with the ASA VR program will promote more positive attitudes and stronger intentions to support people living with dementia. Additionally, it is expected that the knowledge acquired through the accompanying lectures and discussions will enhance health care students’ confidence in delivering care to this population.

This study adopts the conceptual framework proposed by Lane and Yu [20], which identifies three key components essential for fostering dementia-friendly communities: dementia-related knowledge, attitudes toward people living with dementia, and the intention to provide support. The model is grounded in two theoretical assumptions: first, that increased knowledge about dementia is positively associated with a stronger intention to assist people living with dementia; second, that attitudes function as a mediating variable between knowledge and intention, shaping intention and potentially translating into actual supportive behaviors. Thus, the model suggests that enhancing both knowledge and attitudes can increase the intention to offer assistance.

The questionnaire used in this study was originally developed in English and Japanese, has been validated, and has been used in previous studies [15,16]. For this study, it was translated into Indonesian using standard forward-backward translation procedures [21].

#### Attitudes Toward People Living With Dementia

The primary outcome of this study is the attitude toward people living with dementia. Attitude will be measured using the Attitudes Toward People with Dementia Scale [22]. Attitude is understood as a psychological and neurological condition shaped by experience that affects how individuals perceive and respond to objects and situations, thereby guiding or influencing their behavior [23,24]. The stigma associated with dementia represents a critical issue that impacts not only individuals living with the condition but also their families and the wider community. Prevailing negative perceptions and misconceptions within the general public often contribute to experiences of social exclusion, isolation, and discriminatory treatment against those with dementia [25,26]. Stigma continues to serve as a major impediment to the social inclusion of individuals with dementia. Therefore, public awareness initiatives and policy interventions that address discrimination and foster the development of dementia-friendly communities are crucial [27,28]. Attitude has been reported to be the most suitable outcome measure for evaluating the effectiveness of dementia educational programs [29,30].

Attitudes toward people living with dementia will be evaluated using a 14-item instrument developed on the basis of prior studies. The tool uses a 4-point Likert scale ranging from 1 (“strongly disagree”) to 4 (“strongly agree”) and includes 4 subscales: tolerance (5 items), avoidance (4 items), perceived social distance (3 items), and affinity (2 items). The overall score ranges from 14 to 56, with higher scores reflecting more positive attitudes. Tolerance will be measured with statements such as, “I am open to having more interactions with people living with dementia in my daily life.” “Avoidance” will be assessed using items such as, “I prefer to minimize interactions with people living with dementia as much as possible.” Perceived social distance will be captured through items such as, “If a family member were to develop dementia, I would be concerned about others’ opinions.” Affinity will be evaluated with statements such as, “If someone with dementia needs assistance, I would willingly offer help.”

#### Intention to Help People With Dementia

The intention to help was measured using 4 vignettes adapted from prior research depicting situations in which people living with dementia may require assistance. Previous studies indicate that dementia-related education enhances supportive intentions by fostering positive attitudes toward people living with dementia and improving recognition of their assistance needs [31]. These vignettes depict everyday situations in which people living with dementia may require support. For example, 1 vignette describes encountering an unfamiliar older woman in the neighborhood during the summer who is wearing a heavy coat but does not appear distressed. The surroundings are quiet, no bystanders seem concerned, and the participant is not under time pressure. After reading each vignette, participants are asked to respond to the statement *“You will help her”* using a 4-point Likert scale ranging from 1 (“strongly disagree”) to 4 (“strongly agree”). Higher scores indicate a greater intention to help individuals living with dementia.

#### Ageism

Ageism represents broader age-related stereotypes and prejudices toward older adults regardless of cognitive status, whereas attitudes toward people living with dementia focus on dementia-specific stigma and social acceptance. Given the theoretical distinction between these variables and their potential differential impact on dementia treatment, ageism will be assessed independently as a secondary outcome. Participants’ ageist attitudes will be assessed using selected items from the Fraboni Scale of Ageism (FSA). The FSA is a validated tool for measuring stereotypes, prejudice, and discriminatory attitudes toward older individuals [32]. In this study, a subset of key items will be adapted and translated into Indonesian using a forward-backward translation procedure to ensure cultural and linguistic accuracy. The items chosen span 3 major domains: stereotyping, social distancing, and discriminatory views. Each item will be scored on a 5-point Likert scale (1=“disagree“ to 5=“agree”), with higher scores indicating more ageist attitudes. Items that represent positive opinions toward older adults will be reverse-scored (items 13-16).

#### Knowledge of Dementia

Knowledge of dementia will be assessed using the “Dementia Knowledge Scale” by Mikami et al [33]. This scale evaluates understanding of dementia, including its symptoms, treatment, and diagnosis. Respondents will answer using a 3-point Likert scale consisting of “agree,” “disagree,” and “don’t know.” Each item will be scored as 1 for a correct answer and 0 for an incorrect or “don’t know” response. The total score reflects the individual’s level of dementia knowledge, with a higher score indicating greater understanding.

### Data Collection

Participants will be instructed to have a mobile phone or a personal computer ready before attending the class. To complete the online questionnaire, participants must open the provided form, enter the required information, answer all questions, and submit their responses. Access to the questionnaire will not require logging into a Google account; participants will only need to enter their assigned ID number at the beginning of the form. The questionnaire link will be distributed via a QR code displayed on the classroom screen.

### Demographic Data

Participant demographic data will be collected, including age, gender, institutional affiliation, connection to people living with dementia, prior experience supporting and caring for older adults (including those with dementia), and history of attending dementia-related educational sessions, either through formal coursework or through programs offered by dementia-focused organizations.

### Data Analysis

Statistical analyses will be conducted in a stepwise manner. Descriptive statistics will first be used to summarize participants’ demographic characteristics and baseline measures. A complete-case analysis will be used to handle missing data, in which participants with missing data on the primary outcomes will be excluded from the analysis.

To examine within-group changes, 2-tailed paired *t* tests will be conducted to compare preintervention and postintervention scores for attitudes toward people living with dementia, dementia knowledge, ageism, and intention to help. However, the primary analysis will be based on the 2×2 factorial design.

To test the study hypotheses regarding the effectiveness of the ASA VR program, an ANCOVA will be performed for each outcome variable. The dependent variable will be the postintervention score, while the corresponding preintervention score will be entered as a covariate. Two fixed factors will be included in the model: the current intervention condition (ASA VR vs standard course materials) and prior VR exposure (VR-exposed vs non-VR-exposed participants). The model will test 3 effects: the main effect of the ASA VR intervention, the main effect of prior VR exposure, and the interaction effect between the ASA VR intervention and prior VR exposure.

This analysis will determine whether the ASA VR intervention improves attitudes toward people living with dementia, dementia knowledge, ageism, and intention to help; whether prior exposure to VR-based dementia education influences these outcomes; and whether the effect of ASA VR differs between participants with and without prior VR exposure. When significant interaction effects are identified, simple-effects analyses will be conducted to compare the effect of ASA VR within each prior-exposure group. Pairwise comparisons will be adjusted using the Bonferroni correction. Effect sizes will be reported using partial eta squared for ANCOVA effects and Hedges *g* for pairwise comparisons. Statistical significance will be determined using a 2-tailed α level of .05. All analyses will be performed using IBM SPSS Statistics (version 30.0).

### Ethical Considerations

The research protocol for this study received ethical approval from the Ethics Committee of the Faculty of Nursing, Universitas Airlangga (3759-KEPK). All participants retain their unique identification codes from the previous study to maintain confidentiality and enable longitudinal data linkage. Participation remains voluntary, and informed consent will be reaffirmed before data collection begins.

### Dissemination Plan

The findings from this study will be disseminated through peer-reviewed publications, academic conferences, and institutional educational forums. The results are also expected to support the integration of immersive learning technologies into health care education curricula related to dementia education programs in Indonesia.

## Results

Data collection began in December 2025 and was completed in June 2026, with a total of 182 participants recruited. Data analysis is currently ongoing. The effect of ASA VR on attitudes toward people living with dementia, dementia knowledge, ageism, and helping behavior has yet to be determined. The study outcomes will be shared through publication in a peer-reviewed journal.

## Discussion

### Principal Findings

This study is expected to provide important insights into the potential effectiveness of an ASA VR program in improving nursing students’ attitudes toward people living with dementia, dementia-related knowledge, ageism, and intention to help. In contrast to previous VR interventions that relied on passive observation using 360° videos [8], ASA VR is designed as an interactive, scenario-based learning experience that allows students to communicate with AI-driven characters portraying people living with dementia and to make decisions in complex care situations.

This approach operationalizes the principles of problem-based learning and anchored learning, which are effective for enhancing knowledge transfer and developing practical competencies in the field of health education [34]. This study will adopt the conceptual framework proposed by Lane and Yu [20], which identifies knowledge, attitudes, and helping intention as essential components in shaping supportive behaviors toward people living with dementia, with attitudes serving as a mediator between knowledge and intention. By integrating immersive VR experiences with adaptive behavioral simulations, this study is expected to contribute to a deeper understanding of how technology-enhanced learning may influence dementia-related attitudes and behavioral intentions among health care students.

### Comparison With Prior Work

This scenario-driven VR for dementia education is designed to place students in situations that simulate real-life conditions commonly experienced by older adults with dementia. Previous VR-based dementia education studies have demonstrated the potential of immersive learning experiences to improve empathy, dementia knowledge, and attitudes toward people living with dementia [6,35,36]. However, previous interventions primarily focused on passive observation through 360° videos or perspective-taking experiences. In contrast, the ASA VR program extends prior approaches by incorporating AI-based conversational interactions and scenario-driven communication exercises that allow participants to actively practice supportive responses in simulated dementia-care situations.

This study also differs from previous dementia educational interventions by combining immersive VR technology with adaptive communication systems capable of generating context-sensitive interactions. Such an approach may provide a more behavior-oriented educational experience that moves beyond empathy development toward practical communication and supportive behavioral training [37].

### Strengths and Limitations

This study has several strengths. First, to the best of our knowledge, this is among the first AI-based, scenario-driven VR dementia education protocols developed in Indonesia for health care students. Second, the intervention combines immersive VR, adaptive communication, and behavioral simulation within culturally relevant dementia-care scenarios. Third, the factorial design enables examination of both the effects of the ASA VR intervention and the influence of prior VR exposure on educational outcomes.

Nevertheless, this study presents several limitations that should be carefully considered. First, changes in participants’ attitudes, knowledge, or behavior may have been influenced by external factors not accounted for in the study design, potentially confounding the observed results. Second, the sample consisted only of health students from a single educational institution, which may limit the generalizability of the findings to a broader population. To increase the validity and applicability of future research, it is recommended that future studies include a more diverse group of participants and adopt a longitudinal design to assess the sustainability of the intervention’s impact over time.

### Future Directions

Future studies are encouraged to examine the long-term effects of AI-based VR dementia education using longitudinal designs. Further development of the ASA VR program may also include training on nonverbal communication aspects, such as appropriate body posture and maintaining eye-level alignment when interacting with people living with dementia, as well as more adaptive conversational scenarios. Expanding the range of scenarios would allow learners to experience more realistic and dynamic interactions that better reflect real clinical practice. The program may also incorporate interprofessional learning applications and broader implementation among health care professionals, caregivers, and community populations.

### Conclusions

This study introduces a novel approach to dementia education through a scenario-driven VR experience for health care students. By integrating immersive simulation, adaptive communication, and behavioral practice within realistic dementia-care situations, the study is expected to provide insights into the potential role of AI-enhanced VR learning in improving dementia-related attitudes and knowledge and in fostering helping intentions. The findings from this study are expected to advance innovative, culturally relevant educational strategies for dementia awareness and care in Indonesia.

## Supplementary material

10.2196/95684 Peer Review Report 1 The comment and result of peer review.

10.2196/95684 Peer Review Report 2 English translation (AI) of the peer review results.

## Abbreviations

ASA: Awareness Simulation Adventure
FSA: Fraboni Scale of Ageism
LLM: large language model
NPC: non-playable character
TIDieR: Template for Intervention Description and Replication
VR: virtual reality
