# Supplementary material for: Effectiveness of Scenario-Driven Virtual Reality for Improving Nursing Students’ Behavioral Responses Toward Older Adults With Dementia: Protocol for a 4-Arm Postintervention Study
Source: JMIR Res Protoc. 2026 Sep 22;15:e95684. doi: 10.2196/95684 (PMC13596761; doi:10.2196/95684)
Supplement: Supplementary file 1 [file resprot-v15-e95684-s001.pdf]

Detail Penelitian

Scenario-Driven Virtual Reality Game for Dementia Education Among Healthcare Students

← Kembali

Data Litabmas | Bidang dan Luaran | Anggota | Dokumen | Dokumen Luaran | Review

Detail Litabmas

Tahun Sumber Dana	:	2025
Sumber Dana	:	RKI2025 - RISET KOLABORASI INDONESIA
SKIM	:	RKI-C-2025 - SKEMA C
Tahun Aktif	:	2025
Judul	:	Scenario-Driven Virtual Reality Game for Dementia Education Among Healthcare Students
Abstraksi	:	Introduction: The growing number of people living with dementia (PLWD) presents a global challenge. Nursing students require specialized training to provide patient-centered care for PLWD. Virtual Reality (VR) is increasingly recognized in dementia education, offering immersive first-person experiences to enhance empathy among nursing students. Purpose: This study aims to develop a VR-based dementia education program for integration into nursing curricula. Year 1: Develop a VR game, conduct a pilot pre-post study to assess its effectiveness among nursing students, and gather feedback for improvements. Year 2: Refine the VR game, conduct a Randomized Controlled Trial (RCT) for evaluation, and develop a prototype dementia education package for broader implementation. Methods: ?Development: Design and create a VR game for dementia education. ? Implementation: Integrate the program into the Gerontological Nursing Course using Instructional Design (ID), Experiential Learning Model, First Principles of Instruction, and Jasper's Seven Design Principles. ?Evaluation: Assess usability and effectiveness of the program. Subjects & Implementation: Indonesian nursing students will participate in a pre-post study using individual-based allocation with a single-arm sampling approach. The research team will develop the VR game and integrate it into nursing education. Measurement: The primary outcome is attitudes toward PLWD. Secondary outcomes include intentions to help, ageism, dementia knowledge, and VR usability. This study will contribute to the development of structured dementia education programs in Indonesia.
Dana yang diajukan	:	Rp375.000.000,00
Dana yang disetujui	:	-

Copyright © 2026 Institut Teknologi Sepuluh Nopember

Detail Penelitian

Scenario-Driven Virtual Reality Game for Dementia Education Among Healthcare Students

← Kembali

Data Litabmas | Bidang dan Luaran | Anggota | Dokumen | Dokumen Luaran | Review

** Dokumen yang bisa didownload adalah dokumen terakhir yang di upload oleh peneliti*

Dokumen Proposal

Proposal

File: Download

Terakhir diupload: 2025-04-13 13:42:33.873

Dokumen Awal

Buku Rekening
Belum Disetujui

File: Download

Unggah

Dokumen Progress Kemajuan

Laporan Kemajuan

File: Download

Terakhir diupload: 2025-09-01 09:02:26.010

Unggah

Log Book

File: Download

Terakhir diupload: 2025-09-01 09:02:42.887

Unggah

Laporan Keuangan Termin 1

File: Download

Terakhir diupload: 2025-09-01 09:03:05.113

Unggah

Dokumen Progress Akhir

Laporan Akhir

File: Download

Unggah

Log Book

File: Download

Terakhir diupload: 2025-11-21 17:20:04.497

Unggah

Laporan Keuangan Termin 2

File: Download

Terakhir diupload: 2025-11-21 17:20:14.270

Unggah

Copyright © 2026 Institut Teknologi Sepuluh Nopember

Reviewer 1 | Reviewer 2

Detail Penilaian

No	Nama Kriteria	Bobot	Nilai	Total	Komentar
1	Kesesuaian metode penelitian dengan tujuan riset	15	7	105	Metode riset yang meliputi tahap pengembangan, uji kegunaan, dan penilaian efektivitas awal dari program pendidikan demensia berbasis permainan VR sesuai dengan tujuan untuk menghasilkan dan menguji efektivitas permainan VR pada demensia
2	Potensi keberlanjutan hasil penelitian	10	7	70	Riset memiliki potensi keberlanjutan untuk dapat lanjut ke tahap pengembangan teknologi metaverse pada tahun 2027
3	Kontribusi anggota tim riset	20	7	140	Kontribusi/pembagian tugas anggota tim peneliti/mitra sudah dituliskan dengan jelas dan lengkap sesuai bidang keilmuan dan ketersediaan fasilitas di masing-masing lokasi mitra.
4	Potensi ketercapaian luaran	20	6	120	Potensi riset sebagai inovasi metode Baru dalam Program Edukasi Kesehatan Lansia untuk demensia rasional. Namun tidak mencantumkan potensi luaran riset ke jurnal bereputasi (Q1/Q2/Q3)
5	Kepatuhan terhadap ketentuan administratif	10	7	70	Telah memenuhi seluruh poin kriteria/syarat pada Kriteria Penilaian Administratif RKI (Terdapat kesesuaian pengusul dengan dokumen yang diunggah, Host sudah berasal dari salah satu PTNBH, Host sudah berkualifikasi Doktor (S3), Host sudah memiliki H-index Scopus, Host memiliki 3 mitra, Mitra sudah berkualifikasi Doktor (S3), Telah melampirkan Formulir Kesediaan Peneliti Mitra RKI
6	Urgensi masalah yang akan diselesaikan oleh riset yang diajukan	25	5	125	Riset dapat memiliki dampak yang luas bagi masyarakat dan akan menjawab masalah di daerah tertentu. Indonesia belum memiliki program edukasi demensia yang tersusun secara sistematis, namun hanya terbatas pada populasi lansia yang mengalami demensia.
	**TOTAL**			**630**	

Rekomendasi Dana	Rp 375.000.000
Rekomendasi Tahun	1
Komentar	Secara keseluruhan penulisan proposal dalam hal latar belakang, tujuan penelitian, metode yang digunakan, roadmap penelitian dll sudah bagus dan lengkap. Untuk potensi ketercapaian luaran, ini merupakan potensi riset sebagai inovasi metode baru dalam Program Edukasi Kesehatan Lansia untuk demensia rasional. Namun tidak mencantumkan potensi luaran riset ke jurnal bereputasi (Q1/Q2/Q3)

Detail Penelitian

Scenario-Driven Virtual Reality Game for Dementia Education Among Healthcare Students

← Kembali

Data Litabmas | Bidang dan Luaran | Anggota | Dokumen | Dokumen Luaran | Review

Hasil Review

− SELEKSI PROPOSAL

Reviewer 1 | Reviewer 2

Detail Penilaian

No	Nama Kriteria	Bobot	Nilai	Total	Komentar
1	Kesesuaian metode penelitian dengan tujuan riset	15	5	75	sesuai
2	Potensi keberlanjutan hasil penelitian	10	6	60	Berpotensi dalam pengembangan model edukasi inovatif penanganan dimensia
3	Kontribusi anggota tim riset	20	7	140	sesuai
4	Potensi ketercapaian luaran	20	6	120	berpotrnsi bagus untuk publikasi dan luaran video edukasi
5	Kepatuhan terhadap ketentuan administratif	10	7	70	lengkap
6	Urgensi masalah yang akan diselesaikan oleh riset yang diajukan	25	6	150	Urgensi untuk pemecahan masalah dimensia nasional dan internasional
	**TOTAL**			**615**	

Rekomendasi Dana	Rp 375.000.000
Rekomendasi Tahun	1
Komentar	proposal penelitian ini konprehensif dalam menyampaikan program terhadap penangan dimensia nasional dan internasional dan memiliki potensi berkelanjutan

+ MONITORING KEMAJUAN

+ MONITORING AKHIR

Copyright © 2026 Institut Teknologi Sepuluh Nopember
