# Supplementary material for: Effectiveness of Scenario-Driven Virtual Reality for Improving Nursing Students’ Behavioral Responses Toward Older Adults With Dementia: Protocol for a 4-Arm Postintervention Study
Source: JMIR Res Protoc. 2026 Sep 22;15:e95684. doi: 10.2196/95684 (PMC13596761; doi:10.2196/95684)
Supplement: Supplementary file 2 [file resprot-v15-e95684-s002.pdf]

RESEARCH DETAILS

Scenario-Driven Virtual Reality Game for Dementia Education Among Healthcare Students

Research Information
Funding Year: 2025
Funding Source: RKI2025 - Indonesian Collaborative Research
Scheme: RKI-C-2025 - Scheme C
Active Year: 2025

Abstract
Introduction: The growing number of people living with dementia (PLWD) presents a global challenge. Nursing students require specialized training to provide patient-centered care. Virtual Reality (VR) enhances empathy through immersive experience.
Purpose: Develop a VR-based dementia education program integrated into nursing curricula.
Year 1: Develop VR game and pilot study.
Year 2: RCT and program refinement.
Methods: Development, implementation, evaluation.
Participants: Indonesian nursing students.
Outcomes: Attitudes, intention to help, ageism, knowledge, usability.

Proposed Budget: IDR 375,000,000
Approved Budget: -

Proposal Evaluation – Reviewer 2

No	Criteria	Weight	Score	Total	Comments
1	Suitability of research methods with objectives	15	7	105	Methods include development, usability testing, and effectiveness evaluation aligned with objectives
2	Sustainability potential	10	7	70	Potential for continuation into metaverse technology development by 2027
3	Team contribution	20	7	140	Clear division of roles based on expertise and facilities
4	Output achievement potential	20	6	120	Innovative but lacks publication targets in reputable journals (Q1/Q2/Q3)
5	Administrative compliance	10	7	70	All administrative requirements fulfilled
6	Problem urgency	25	5	125	Broad impact; Indonesia lacks structured dementia education programs
	TOTAL			630	

Funding Recommendation: IDR 375,000,000
Year Recommendation: 1
Comment: Proposal is comprehensive but lacks clear publication targets.

Proposal Evaluation – Reviewer 1

No	Criteria	Weight	Score	Total	Comments
1	Suitability of methods	15	5	75	Appropriate
2	Sustainability potential	10	6	60	Potential for innovative dementia education models
3	Team contribution	20	7	140	Appropriate
4	Output potential	20	6	120	Good for publication and educational video outputs
5	Administrative compliance	10	7	70	Complete
6	Problem urgency	25	6	150	Important nationally and internationally
	TOTAL			615	

Funding Recommendation: IDR 375,000,000
Year Recommendation: 1
Comment: Comprehensive proposal with strong national and global relevance and sustainability potential.
